# Further Remarks on Pyorrhœa Alveolaris

**Published:** 1894-08

**Authors:** C. N. Peirce

**Affiliations:** Philadelphia


### FURTHER REMARKS ON PYORRHŒA ALVEOLARIS.[1]

BY C. N. PEIRCE, D.D.S., PHILADELPHIA.

In two previous communications, one published in the International Dental Journal for January, 1894, the other in the *Dental Cosmos* for February, 1894, there are stated, with some detail, views which I entertain, and which appear to me to be sustained by recent experience, regarding the etiology, pathology, and treatment of pyorrhœa alveolaris.

In these two papers facts and deductions are presented which, if rightly interpreted, it is believed will establish the kinship of pyorrhœa, or as previously designated, hæmatogenic pericementitis, with the gouty or uric-acid diathesis.

The conclusions entertained may be well represented in a condensed form in the following postulates,—viz.:

1. The inflammatory stage of true pyorrhœa alveolaris primarily begins in tissues on the side of the root near the apical extremity, and secondarily advances in the very large majority of cases towards the gingival borders.

2. The cause of this inflammation, or gingivitis and pericementitis, is the plasma exudation from the blood-vessels freighted with salts, which, in their deposition and crystalization upon the cementum of the root and infiltration of the more vascular tissues, exert the influence of foreign bodies and react as irritants.

3. The salts in question, as disclosed by chemical analysis, are calcium and sodium urates, free uric acid, and calcium phosphate.

4. The chemical nature of these salts indicates a condition of the blood in which there is an excess of uratic salts and uric acid, due to either *increased formation* or *imperfect elimination.* The excess of these salts, as is well known, is regarded by general pathologists as indicative of a faulty nutrition, and is the immediate cause of a series of local disturbances to which the term gouty has been applied, the nutritional disturbance being known as the uric-acid diathesis.

5. An attentive study and accurate observation of the various organs and tissues of patients suffering with pyorrhœa alveolaris have disclosed the co-existence, in a very large proportion of them, of one or more local expressions of this constitutional diathesis.

---

[1] Read before the Odontological Society of Pennsylvania, March 10, 1894.

6. Recognition of the fact that a constitutional malady presents itself, one phase of which only has claimed the attention of the dental practitioner, indicates that a treatment designed to be curative must have reference not only to the local expression, but especially to this important systemic condition as well.

7. Results from constitutional treatment in connection with the usual local applications in a number of well authenticated cases of pyorrhœa have been so markedly satisfactory that the writer feels fully justified in his assumptions regarding the origin of the disease.

An objection that might be and, indeed, has already been urged to these conclusions is, that while some of the cases of pyorrhœa might be considered as either the local expressions of a constitutional condition, or the sequence of a specific poison acting as a predisposing cause, yet a large percentage, it is claimed, cannot be so estimated. *The force of this objection must depend entirely upon what conditions are meant to be represented by the term pyorrhœa alveolaris.*

In a former communication an attempt was made to differentiate two distinct diseases of the pericemental membrane, both of which were caused by the deposition of salts. In this short paper I desire to recognize the fact that we have *three* distinct conditions, quite easily differentiated, which it appears are not infrequently—indeed, are generally—classed under the same heading, "Pyorrhœa Alveolaris." The *first* may be noted as inflammation and destruction of the gum-tissue and the pericemental membrane, with solution of the alveolar process,—this the result of a mechanical irritant, the salivary calculus. This may or may not be accompanied by a flow of pus. If the pus be present, it is dependent upon some constitutional predisposition such as a scorbutic or scrofula habit. *Second.* Inflammation of the gingival borders with a varying flow of pus, but without the presence of tartar,—phagedenic gingivitis and pericementitis,—so designated by Dr. Black. This is greatly exalted, if not wholly caused, by some morbid constitutional state, which is also a destructive process. *Third.* Alveolo-cemental irritation originating at or near the apical extremity of the root, and due to some morbid composite of the blood exuded with the plasma, a salt or salts, infiltrating this locality of the membrane and *usually* becoming precipitated upon the root of the tooth near the apex; this latter I designate true pyorrhœa alveolaris, or hæmatogenic pericementitis. It is accompanied by accumulation and flow of pus, by denudation of a portion, or all, of the root, by dissolution of the process, by turgescence of the gum, and in certain stages with considerable pain. This inflammatory condition, with its concomitants, being so invariably associated with some recognized manifestation of a gouty diathesis, there appears to be full warrant for stating that it is but another local expression of that systemic condition. Confirmation of this assumption is found in the fact that this accumulation which is so frequently recognized upon the root near its apical extremity has by analysis been proved to consist of such foreign bodies as sodic urate, calcic urate, and free uric-acid crystals. Nor is the presence of these salts the only evidence of the correctness of this theory. Cases which have been locally treated for months, indeed for years, with only a very limited perceptible improvement, have, when the patient has been subjected to constitutional treatment in addition, responded promptly; the inflammatory conditions, with their progressive concomitants, pus and process absorption, subsiding in a great degree. It is a matter of almost daily occurrence and observation, that a chronic latent inflammation of the pericementum may undergo an exacerbation from mechanical or atmospherical causes from the accumulation of the product of decomposition in the tooth, which may be followed by the formation of pus. This form of pericementitis may be of long or short duration, but in most every instance it yields promptly to local treatment. Now, although this inflammation be attended with a flow of pus and much suffering, it is in no wise a specific inflammation, and frequently occurs in individuals in whom no trace of a gouty tendency could be detected. And when the train of symptoms of such an inflammation are contrasted with those of the disease with which all are familiar, and which is known, or has been known, as Riggs's disease and pyorrhœa alveolaris, there can be no doubt that two distinct conditions are to be diagnosticated and treated. It is conceivable, however, that a local, non-specific, easily controlled pericementitis might, by the deposition of uric acid, become a specific, persisting, and destructive pericementitis. It is then this phase of the disease, whether *primary* or *secondary*, to which is applied the term hæmatogenic, and which is to be regarded as a local manifestation of a constitutional condition.

In the remaining moments devoted to this paper an effort will be made to outline a course of treatment based wholly on the supposition that in its etiology and pathology pyorrhœa as above defined is but one of the multiform phases of the uric-acid diathesis. This, then, very naturally resolves itself into both local and constitutional treatment; the local directed towards the removal of the deposit, and the control or suppression of the inflammation and its concomitants. The method by which this is accomplished has been so frequently described and discussed that further amplification of it in this place seems unnecessary. There is, however, one factor which might be regarded as protective or prophylactic, and, though local, is of considerable moment and deserving of more than a passing notice,—I allude to the exercise of care in the avoidance of injuries to the pericemental membrane. Whatever the predisposing cause may be, the immediate or exciting cause must ever be borne in mind. This, it is believed, to a certain extent at least, is found in all those mechanical agencies so well known to the dentist, which impair or lower the nutritional level of the pericementum, thus rendering it liable, under certain systemic conditions, to a deposition of uratic salts. The question has been raised as to why the membrane of one or more teeth widely separated or occupying positions on opposite sides of the mouth, either simultaneously or successively, become the seat of inflammation when there is no continuity of structure; the answer to this must be found in the fact that impaired nutrition and lowered vitality in such structures are due in the majority of instances to mechanical injury. It is certainly within the experience of many observant dentists that pyorrhœa has not infrequently developed around a tooth after it has been subjected to the necessary mechanical manipulations incident to tooth protection—tooth preservation. This apparent interference with the nutrition of the pericemental membrane before the deposit of uric-acid salts takes place is in accordance with what is believed to hold true for other manifestations of the gouty diathesis. It is generally conceded by pathologists that no tissue becomes the seat of any specific inflammation without some previous alteration in its nutrition, which renders it more or less vulnerable to the morbid agent circulating in the blood. It is well known that this is especially true of acute gout. All the parts exposed to strain, pressure, blows, contusions, etc., such as the joints of the feet and hands, are particularly liable to become the seat of the specific deposit. As a prophylactic measure, therefore, it is suggested that whenever there is the slightest tendency to pyorrhœa, or any other evidence of the gouty diathesis, great care should be exercised in all dental operations, so as not to impair the nutrition of the peridontium and thus establish the necessary condition for the uric-acid deposit. The constitutional treatment which has been indicated as efficient in the elimination of already established uric-acid conditions, and the restoration of a faulty nutrition to its normal state, may with great propriety be subdivided into hygienic and medicinal. The hygienic treatment embraces systematic out-door exercise, stimulation of the functional activity of the excretory organs, such as the skin, bowels, and kidneys, and regulation of the diet. Every dentist must appreciate the value of open air exercise, and if the welfare of the patient is to be considered paramount, it must be insisted upon in all well-marked cases, and especially with those who, for various reasons, lead sedentary and inactive lives.

Increased muscular activity quickens circulation, induces deeper and fuller respiratory movements, leads to greater vigor in the general nutritive processes; waste products are removed more rapidly, and the combustion of the foods increased by the absorption of a large amount of oxygen. The promotion of the functional activity of the eliminating organs is well recognized as an important hygienic measure.

My friend, Professor Brubaker, suggests that where there is need of hygienic treatment the secretory function of the perspiratory and sebaceous glands and the capillary circulation should all be stimulated by sponging of the skin with cold water, vigorous friction, and an occasional Turkish bath, where such treatment is not contraindicated by pulmonary or cardiac affections. And that where the liver and intestinal glands are deficient in secretion with prevailing constipation, they should be stimulated into activity by the use of saline waters; most excellent for this purpose being the Hunyadi Janos and Friedrickehalle. These, he says, are especially to be commended because they contain a large percentage of sodium and magnesium sulphates, both of which are useful as eliminating agents. The kidneys should be assisted in the excretion of waste products by the free use of negative waters, or waters in which the saline constituents are at a minimum quantity. Hot or distilled water in sufficient quantity will flush the alimentary canal, increase the volume of blood, and stimulate the kidneys to increased activity. It is not only of common observation, but rather a remarkable fact, that gouty patients are inclined to drink but a comparatively small quantity of water. One quart of hot water taken daily, in four doses, before breakfast, between meals, and at bed time, is considered most beneficial in its effects in dissolving and removing irritating products. The most important of the hygienic measures in the treatment of all gouty manifestations is that pertaining to the diet. As uric acid is considered a nitrogenized compound, and therefore presumably one of the imperfectly oxidized products of albuminous or nitrogenized foods, it is desirable that such foods be excluded as far as possible from the daily diet. The value of this measure is admitted and insisted upon by all clinicians.

In the milder manifestations of the gouty diathesis, such as we assume exists in pyorrhœa, it is not so imperative that all albuminous foods be prohibited; nevertheless, as many of our patients are consumers of large quantities of meat, it would be well to insist, if the effort to cure is to be made, upon the total exclusion of beef, veal, mutton, and pork, restricting the patient in albuminous diet to white meat of chicken, fish, oysters, and lobsters. Cheese, beans, and the white of eggs are considered objectionable, and in many cases of acute gout are strictly prohibited by the attending physician. Experience has shown that various alcoholic drinks, such as port, madeira, and sherry, are particularly liable to give rise to the accumulation of uric acid. The lighter wines, as claret and hock, are not considered so injurious. The malt liquors,—beer, ale, and porter,—are also, with many clinicians, considered in their influence gross offenders.

If this assumption regarding pyorrhœa alveolaris be correct, the following, also furnished me by Professor Brubaker, is pertinent and applicable: The medical and constitutional treatment—it is obvious—should be directed towards the elimination of uric acid and its compounds. For this purpose remedies which promote the formation of soluble and easily diffusible products which are readily eliminated by the kidneys are indicated. From time immemorial the alkalies and alkaline combinations have been used with marked success in the management of all phases of the gouty diathesis. The treatment of acute gout necessitates, of course, different or more vigorous remedies than those required for the subacute or chronic forms with which the dental practitioners will be called upon to deal. Of the various alkalies, lithium compounds—the citrate and carbonate—have been found best adapted to the milder phases of the disease. The form most easily to take is the compressed tablets containing five grains each of the citrate; one tablet three or four times daily will be found sufficient. Should this not agree with the patient, the potassium carbonate in ten-grain doses, in some simple bitter,—gentian or quassia water,—three or four times daily, can be substituted. A valuable adjunct to the medicinal treatment is the free use of alkaline waters, which assist in the elimination of waste products, though it is probable that the good effects attributed to these are largely due to the quantity of liquid consumed. The Saratoga, Vichy, alkaline waters of Wisconsin, the Marienbad, Carlsbad, Appolinaris, etc., have all been found efficacious. Should the patient be highly dyspeptic, as is frequently the case, remedies directed to the digestive viscera are of course indicated. If anæmia be a concomitant, iron and quinine will be necessary. A combination which has been found of great value in improving the quality of the blood is one of iron and a salt of potassium. Blaud's pill, consisting of these two ingredients, is a desirable form for administration; one three times a day will be sufficient. However ingenious our interpretation of pathological conditions may be, and however plausible our deductions may appear, the ultimate test of their value will be the readiness with which they yield to and disappear under appropriate treatment. If pyorrhæa alveolaris be a manifestation of the gouty diathesis, and the symptoms and pathological conditions which characterize it be excited and maintained by the deposit and pressure of uric acid and its salts, it should be amenable to the same therapeutic measures which have been efficacious in the treatment of all other forms of gout in other portions of the body. It must be borne in mind, however, that though a case be cured for a period of six months, or even a year, this does not preclude a relapse should the patient return to an improper diet or irregular mode of life. It is hardly necessary to say that this is true of all chronic diseases. In individuals predisposed to uric-acid accumulations, an entirely new mode of life is to be instituted and followed with extreme care for a long period of time.

As corroborative, however, of the facts which have been adduced in this and previous papers, in relation to age, occupation, heredity, constitutional condition, as well as the effects of treatment, I append the history of a few cases which may be of interest.

Case I.—Mr. A., aged forty-five. Family history: father died early from consumption; mother rheumatic; has lost nearly all her molars from pyorrhœa, which she has had for many years. The patient has been suffering from pyorrhœa for six or eight years. For nearly three years he has been under my care, his visits being monthly. Though not a tooth has been lost, the thirty-two have been the seat of well-defined pyorrhœa; gums œdematous, with veins engorged, giving a purplish color. Notwithstanding the continued local treatment, the most that can be said as to the benefit derived from this is, that the disease did not progress, nor did it materially improve, save some abatement in the inflammatory condition of the gums. As an evidence of a gouty constitution the patient had suffered with neuralgic headaches, vague rheumatic pains, and great mental depression. Several years ago he was informed by a distinguished clinician that the heart was depressed in its action from nitrogenized waste products. The patient admitted having largely confined his diet to meats. After, now, a little over three months' treatment, embracing restricted diet and the use of lithium and hot water, the discharge of pus has ceased from all the teeth except one third molar. The loosened teeth have become much firmer, the turgid condition of the gums has disappeared, and the gingival borders present a much more normal appearance. The patient on his last visit stated that mastication was performed without fear of dislodging the teeth.

Case II.—Mrs. B., aged fifty-five. Family history, gouty; one sister almost disabled from deposits in joints of hands, feet, and limbs; another sister has lost by erosion the enamel from the six anterior superior teeth. The patient has had pyorrhœa for eight or ten years, from which several teeth have been lost; ten others are now affected with looseness and some discharge of pus. The members of this family have been large consumers of meat. Treatment for two months with drinking hot water and modified diet gave improvement in flow of pus and inflammatory condition of gums.

Case III.—Mrs. C. Lost superior teeth from pyorrhœa; ten teeth remaining in lower jaw, all more or less affected with pyorrhœa. The patient has been under treatment for gout for some years,—all the joints of fingers enlarged and painful from gouty deposits. The forehead and face show the result of herpes from previous herpatic eruption, regarded as of gouty origin. Treatment was followed by improvement.

Without wearying you with details of similar cases, permit me to say that in addition to the three cases just noted, ten others have been, and are, under treatment, all of whom, with the exception of three school-teachers, have presented other manifestations of gout. In these three cases a sedentary life and concentrated nitrogenous food have probably been instrumental in establishing the systemic condition leading to pyorrhœa. It is gratifying to recognize the fact that cases which have been brought under treatment of the restricted diet and the indicated constitutional treatment have given such encouragement that it is not unreasonable to hope that, with a continuation of the therapeutic measures and careful diet, the not-distant future will witness such abatement of the destructive symptoms that the cases can, with few exceptions, be recorded as confirmatory of the theory suggested in the foregoing papers.

The writer can certainly state that the local treatment alone which had previously been employed by him has not yielded such satisfactory results.

